# Prevalence of Common Clinically Manifested Developmental Anomalies of the Oral Cavity Among Adults – An Epidemiological Study in a South Indian Population

**DOI:** 10.7759/cureus.9961

**Published:** 2020-08-23

**Authors:** Lokesh Sundaram, Vidhya Rathnavelu, Divyambika C Venugopal, Malathi Narasimhan, Mythili Sabesan, Swarna Swetha

**Affiliations:** 1 Dentistry, Sri Ramachandra Institute of Higher Education and Research, Chennai, IND; 2 Oral Pathology and Microbiology, Sri Ramachandra Institute of Higher Education and Research, Chennai, IND; 3 Oral Medicine and Radiology, Sri Ramachandra Institute of Higher Education and Research, Chennai, IND; 4 Oral Pathology, Sri Ramachandra Institute of Higher Education and Research, Chennai, IND

**Keywords:** developmental anomalies, oro-facial problems, inheritance, mutations, prevalence

## Abstract

Introduction

Developmental anomalies are malformation which arises due to the disturbances during the development of the organs. Although there have been many studies that have described the prevalence of these anomalies in the oral cavity, none have specified the prevalence of clinically manifested anomalies and their distribution between gender.

Materials and methods

In this study, 500 patients aged 18 to 50 years were screened for clinically manifested developmental anomalies. These were then divided based on age, sex, and jaws, which were then analyzed using a chi-square test and tabulated.

Results

We detected anomalies in 12.2% of the study population. Supernumerary teeth were the most prevalent anomaly detected (4.25%). The frequency of developmental anomalies was higher in men (57.1%).

Conclusions

Supernumerary teeth were the most widely recognized anomaly. Other anomalies identified were related to the shape and size of teeth. These anomalies can lead to severe orofacial problems. Therefore, proper care of these anomalies should be taken.

## Introduction

Developmental anomalies comprise a wide range of abnormalities of body structures or functions and are present at birth or of parental origin [1]. The causes of the appearance of these defects are many. They can be genetic factors such as inheritance, mutation, or different errors at the hereditary level. Likewise, they can be ecological factors that incorporate physical, chemical, or organic elements that cause changes in genes, leading to altered signaling, resulting in the manifestation of developmental anomalies [2,3]. Irregularities relating to tooth size and shape result from disturbances that happen in the morpho-differentiation phase of fetal development. Other defects, like ectopic eruption or impaction, are a result of developmental disturbances that have occurred in the eruption sequence of permanent dentition [4-6].

The degree of prevalence of developmental anomalies of the oral cavity, or any developmental anomalies, may vary with race, gender, and age [6-11]. Numerous pieces of research have proven this. Examples such as talons cusp and peg laterals are more common in permanent teeth than in primary teeth. Supernumerary teeth were more common in whites than in blacks, and anomalies such as talons cusp and supernumerary teeth are more common in men [9-15]. However, these anomalies may be present as an individual trait or in association with other conditions. Syndromes such as micrognathia and U-shaped cleft palate are often associated with Pierre-Robin Syndrome. Microdontia and peg laterals are often associated with Saethre-Chotzen Syndrome, and supernumerary teeth are associated with Gardner’s Syndrome [16,17].

Although research has described the prevalence of oral developmental anomalies, none has specifically investigated clinically manifested developmental anomalies and their distribution among gender. Hence this study aims to establish more reliable data of the prevalence of clinically manifested developmental anomalies for the cognizance of the society under the following criteria: one, to evaluate the most prevalent developmental anomaly; and two, to evaluate the frequency of prevalence of developmental anomalies between gender.

## Materials and methods

This epidemiological study was conducted in the Department of Oral Medicine and Radiology, Faculty of Dental Sciences, Sri Ramachandra Institute Of Higher Education and Research, after obtaining ethical clearance from the Institutional Ethics Committee of Sri Ramachandra Institute of Higher Education and Research.

The study population included patients aged 18 to 20 years. Patients excluded from the study were those who had systemic medical conditions and those who needed emergency treatment.

The sample size was estimated by the use of the Leslie Fischer’s formula for study populations of more than 20,000 at a 95% confidence level with a 50.0% prevalence and a degree of error set at 0.05; the sample size estimate was 323, but the sample size was increased to 500 patients.

Data were collected via clinical examination. The anomalies included supernumerary teeth (i.e., the presence of additional teeth that can be present anywhere in the oral cavity), talon cusps (i.e., a cusp-like projection in the lingual surface of anterior teeth), peg laterals (i.e., the lateral labial surfaces of maxillary lateral incisors are peg-shaped), paramolars (i.e., supernumerary molars present buccally or lingual to molars), mesiodens (i.e., supernumerary incisors present between maxillary central incisors), microdontia (i.e., the size of the teeth is smaller than the prescribed size) [18].

Intra-examiner accuracy testing was performed to calibrate the principal investigator on the consistency of diagnosis for these developmental anomalies. The test was done by examining the clinical photograph of various clinically manifested developmental anomalies. The scoring for each of the anomalies identified correctly was recorded. The intra-examiner accuracy score was sufficiently high.

**Figure 1:**
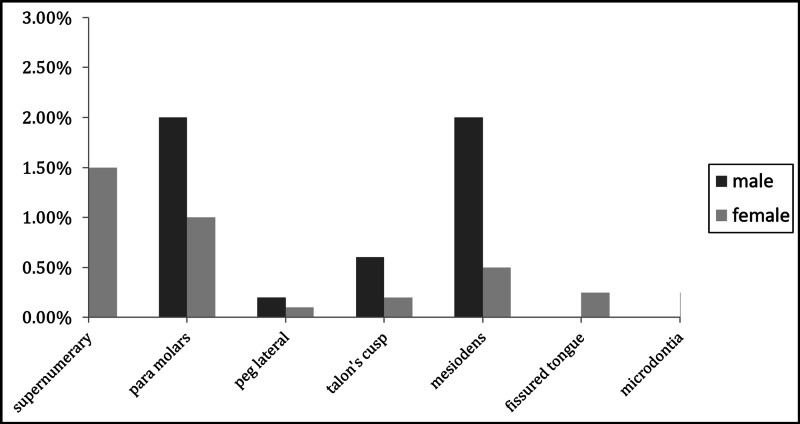
Distribution of anomalies among genders

The collected data were tabulated based on sex, age, and jaw variance using the chi-square test. These data were then compiled into Table 1 and Figure 1.

**Table 1:** Prevalence of developmental anomalies in a population of 500 patients

Variable	Frequency
Gender	
Men	57.1%
Women	42.9%
Age Group	
18-28 years	36.7%
28-38 years	46.9%
38-50 years	16.3%
Anomalies	
Total Anomalies Detected	12%
Supernumerary teeth	4.25%
Paramolars	3%
Peg laterals	1.2%
Talons cusp	0.8%
Mesiodens	2.5%
Microdontia	0.25%

## Results

**Figure 2:**
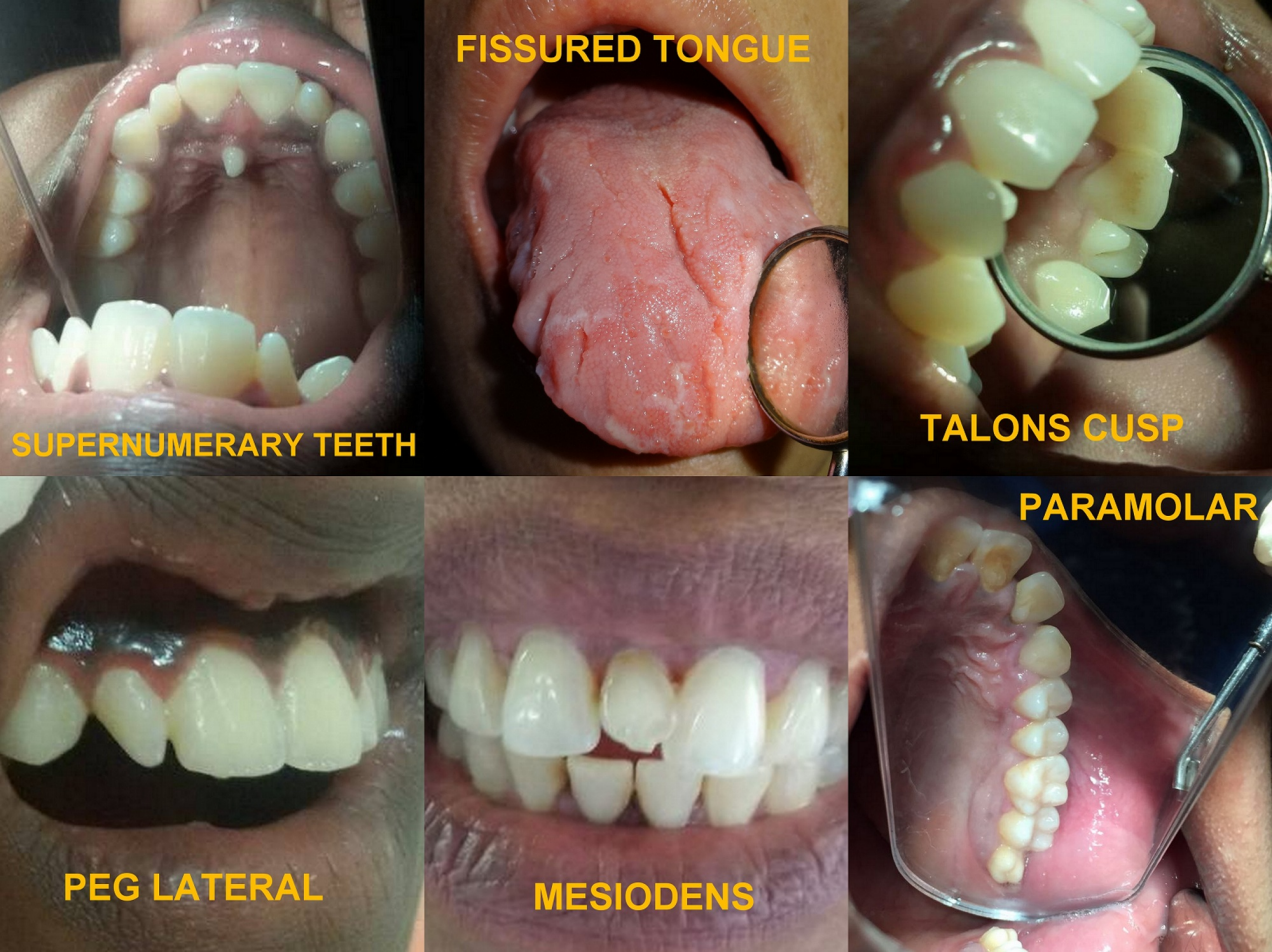
Photographic representation of the developmental anomalies

From the clinical study of 500 patients, it was noted that 12.2% (Table 1) of the study population were detected with anomalies. Of these, 57.1% were men. A detailed listing of the results is given in Table 1. Clinical photographs of the anomalies seen are provided in Figure 2.

A graphical representation of the percentage of orofacial developmental anomalies is shown in Figure 1.

## Discussion

Developmental anomalies of the oral cavity are mainly congenital defects that often compromise the function of the oral cavity and the aesthetics of an individual. We, as health care providers, should identify these anomalies as early as possible so that functional and aesthetic rehabilitation of the dentition can be established early. Through this study, we aimed to know the prevalence of clinically manifested anomalies in a south Indian population for the future development and welfare of the society.

In our study, the prevalence of anomalies was 12.2%. Of these, 57.1% were men, and 43.9% were women. These results match with results reported by Guttal et al. (11%) and Gupta et al. (12%) in studies conducted during 2010 and 2011, respectively [6,13].

The most prevalent anomaly found was supernumerary teeth (4.25%); other frequently found anomalies were paramolars and mesiodens. These findings are in keeping with the results of other studies by Ooshima et al. in Japan and Mohanty et al. in the United States [9,19]. Research by Hamasha et al. who carried out similar epidemiological studies, revealed that certain anomalies like microdontia and talons cusp showed the least prevalence among all other anomalies [20]. This also fell in line with our research.

Several factors contribute to developmental anomalies of the tongue. For example, exposure of pregnant women to environmental factors like teratogenic drugs and radiation, consumption of high fluorine diet, smoking, and alcohol may increase the risk of developmental tongue anomalies in newborns. When environmental factors impact pregnant women, it has been demonstrated to cause developmental anomalies of the oral cavity, specifically those pertaining to the size and shape of the teeth in progeny [21-24].

The higher prevalence of tongue developmental disorders in men is often due to the presence of one sex-linked X-chromosome [25]. This study also noted that the number of anomalies recorded found in maxilla exceeded the number of anomalies detected in the mandible (67.3% in the maxilla and 32.6% in the mandible).

## Conclusions

In spite of being asymptomatic, these developmental defects can lead to serious problems including delayed, no eruption, or impaction of the supernumerary or normal teeth (or tooth); attrition; reduced aesthetics and tongue space; temporomandibular joint discomfort, malocclusion, changes in face profile, and also increase the risk of caries and periodontal issues. It is likewise the case that supernumerary teeth, with a sharp incisal or occlusal tip, can cause constant irritation that can lead to malignant transformation of the mucosa. Therefore, proper care of these anomalies should be taken in order to avoid serious orofacial problems. In this study, we were able to educate patients with anomalies about the long-term effects of these anomalies on their body and systemic functions.
